# A SYSTEMATIC REVIEW AND META-ANALYSIS OF AGE-RELATED DIFFERENCES IN TRUST

**DOI:** 10.1093/geroni/igz038.1775

**Published:** 2019-11-08

**Authors:** Phoebe E Bailey, Tarren Leon

**Affiliations:** Western Sydney University, NSW, Australia

## Abstract

This systematic review and meta-analysis quantifies the magnitude and breadth of age-related differences in trust. Thirty-eight independent data sets met criteria for inclusion. Overall, there was a moderate effect of age group on trust (g = 0.22), whereby older adults were more trusting than young adults. Three additional meta-analyses assessed age-related differences in trust in response to varying degrees of trustworthiness. This revealed that older adults were more trusting than young adults in response to neutral (g = 0.31) and negative (g = 0.33), but not positive (g = 0.15), indicators of trustworthiness. The effect of age group on trust in response to positive and neutral cues was moderated by type of trust (financial vs. non-financial) and type of responding (self-report vs. behavioral). Older adults were more trusting than young adults in response to positive and neutral indicators of trustworthiness when trust was expressed non-financially, but not financially. There was also an age-related increase in self-reported, but not behavioral, trust in response to neutral cues. Older adults were more trusting than young adults in response to negative indicators of trustworthiness regardless of the type of trust or type of responding. The reliability of information about trustworthiness (superficial vs. genuine cues) did not moderate any effects of age on trust. Implications of these findings and directions for future research are discussed.

